# Outcomes of Patent Foramen Ovale Transcatheter Closure: Should a Short Aortic Rim Preclude Closure?

**DOI:** 10.1016/j.jacadv.2023.100257

**Published:** 2023-03-22

**Authors:** Ada C. Stefanescu Schmidt, Lusine Abrahamyan, Annamalar Muthuppalaniappan, Ricardo Gorocica Romero, Georges Ephrem, Karl Everett, Douglas S. Lee, Mark Osten, Leland N. Benson, Eric M. Horlick

**Affiliations:** a Toronto Congenital Cardiac Centre for Adults, Peter Munk Cardiac Centre, University Health Network, Toronto, Ontario, Canada; b Heart Center, Massachusetts General Hospital, Harvard Medical School, Boston, Massachusetts, USA; c Toronto General Hospital Research Institute, University Health Network, Toronto, Ontario, Canada; d Institute of Health Policy, Management and Evaluation, University of Toronto, Toronto, Ontario, Canada; e Toronto Health Economics and Technology Assessment (THETA) Collaborative, Toronto General Hospital, Toronto, Ontario, Canada; f Gleneagles Hospital Penang, Pulau Pinang, Malaysia; g Mexican Institution of Social Security, UMAE No. 1, Merida, Yucatan, Mexico; h Krannert Institute of Cardiology, Indiana University School of Medicine, Indianapolis, Indiana, USA; i ICES, Toronto, Ontario, Canada; j Division of Cardiology, The Hospital for Sick Children, Toronto, Ontario, Canada

**Keywords:** aortic rim, atrial septal defect, embolization, patent foramen ovale, transcatheter device closure

## Abstract

**Background:**

The risk of erosion of an atrial septal closure device, in particular the Amplatzer Septal Occluder, has been described as higher in patients with a short aortic rim. Similar concern has been applied to patent foramen ovale (PFO) closure devices, but there are only rare reported cases of erosion. It may be that smaller devices are chosen due to fear of device erosion in PFO patients when this is not necessarily an issue.

**Objectives:**

The authors aimed to assess outcomes after PFO closure with the Amplatzer PFO device in patients with a short (<9 mm) aortic rim.

**Methods:**

We performed a retrospective analysis of PFO closure for any indication, between 2006 and 2017 at a quaternary center. Preprocedural transesophageal echocardiographic parameters including the aortic rim were remeasured. Long-term outcomes were obtained by linkage to provincial administrative databases.

**Results:**

Over the study period, 324 patients underwent PFO closure with the Amplatzer PFO device, with a mean age of 49.8 years; 61% had a short aortic rim (<9 mm). The most common indication was cryptogenic stroke (72%); those with longer aortic distance were more likely to have a non-stroke indication for closure, diabetes (15% vs 6.5%, *P* = 0.04), and heart failure (15.7% vs 4%, *P* < 0.001). Over a median 7 years of follow-up, there were no cases of device erosion or embolization requiring cardiac surgery.

**Conclusions:**

In a large cohort with long-term administrative follow-up (1,394 patient-years), implantation of an Amplatzer PFO device was performed safely even in patients with a short aortic rim.

Percutaneous patent foramen ovale (PFO) closure is now supported by guidelines in highly selected patients with cryptogenic stroke, after thorough evaluation by a neurologist.^1^, ^2^, ^3^ Less common reasons for closure include platypnea-orthodeoxia syndrome, prophylaxis of decompression illness for scuba diving,^4^ and prevention of paradoxical embolism in selected patients with permanent indwelling lines or pacemaker wires. The incidence of procedural complications in clinical trials and registries is very low, with a 0.02% to 0.2% incidence of device erosion^5^ or cardiac perforation^6^ in long-term follow-up, with an additional 1.4 per 100 person-years incidence of new atrial fibrillation,^7^ and 0.58 per 100 person-years incidence of recurrent stroke.^6^

The Amplatzer PFO Occluder (Abbott Structural Heart) and Gore Cardioform (W.L. Gore) are both approved for use in the United States and Canada. The initial instructions for use, as included in the Federal Drug Administration approval in 2016, note that the Amplatzer PFO Occluder should only be implanted in patients with adequate aortic and superior vena cava rims, defined as 9 mm on echocardiography.^8^ The PFO to aortic distance is however shorter than that for many adults.^9^ These instructions for use were updated in 2019, and now state that the rims have to be ‘adequate’ without specifying a measurement^10^; for the Amplatzer Septal Occluder, caution is recommended in patients with deficient rims (<5 mm).^11^ Unlike for the Amplatzer Septal Occluder,^12^ there is a paucity of data to support a higher risk of embolization or erosion of the PFO occluder in patients with a short aortic rim. Theoretically, the structural characteristics of the PFO device, as it is more flexible, not self-centering, and both right and left atrial discs are significantly larger than the defect, may reduce the risks of erosion and embolization compared to the Amplatzer Septal Occluder. In the RESPECT (Randomized Evaluation of Recurrent Stroke Comparing PFO Closure to Established Current Standard of Care Treatment) trial, there were no cases of device embolization reported in the short- or long-term follow-up and only one case of cardiac perforation in the 499 subjects in the device group (further details not available).^6^^,^^13^ There are however 3 cases reported in the literature of erosion with the Amplatzer PFO device, of which one was within the first 24 hours of the procedure, and the 2 others at 11 and 16 months after closure.^14^, ^15^, ^16^

We sought to compare the incidence of procedural and long-term adverse events after percutaneous PFO closure with the Amplatzer PFO Occluder by aortic rim distance (ie, <9 mm vs ≥9 mm). We hypothesize that the Amplatzer PFO Occluder does not require a minimal aortic rim for safe implantation.

## Methods

### Study population

A retrospective chart review of all PFO closures performed at the Peter Munk Cardiac Center (Toronto General Hospital, Toronto, Canada) between 2007 and 2017 was performed. Patients were candidates for PFO closure after a cryptogenic stroke or for a non-stroke presentation such as decompression illness during scuba diving, indwelling right heart lines or pacemaker, and platypnea-orthodeoxia syndrome. Patients with cryptogenic stroke were evaluated by a neurologist for other possible causes of stroke, and had magnetic resonance imaging of the brain, imaging of the aorta and head and neck vessels, at least a 48-hour monitor to evaluate for atrial arrhythmias (most patients had at least 2 weeks of monitoring as practice evolved), and a transesophageal echocardiogram (TEE) to evaluate for source of embolism. Presence of a PFO and right to left shunting with a positive bubble study was confirmed by TEE prior to the procedure.

Demographics, imaging and procedural characteristics, and periprocedural outcomes were abstracted from the chart. Procedural success was defined as device implantation without retrieval or death. Periprocedural outcomes were defined as occurring prior to hospital discharge.

### Echocardiographic analysis

**Figure 1:**
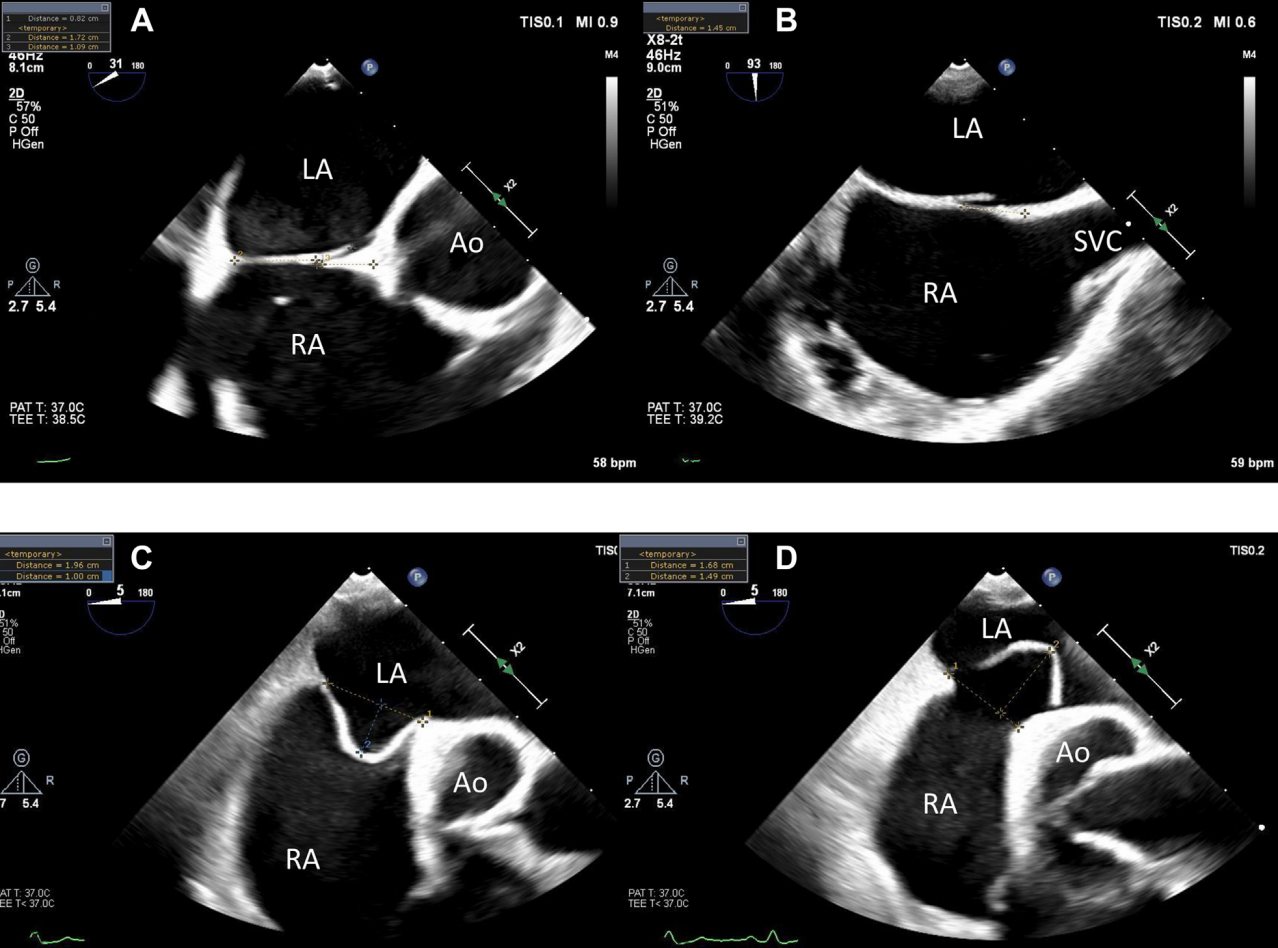
**Measurement of Aortic and Superior Vena Cava Distance, and Atrial Septal Excursion by Transesophageal Echocardiogram** **(A)** Mid-esophageal 30° view, with measurement of the posterior and aortic rim. **(B)** Mid-esophageal 90° view, with measurement of the superior vena cava rim. **(CandD)** Mid-esophageal 0° to 5° view of an aneurysmal atrial septum, with measurement of septal excursion into the right **(C)** and left **(D)** atria. Ao = aorta; LA = left atrium; RA = right atrium; SVC = superior vena cava.

Available preprocedural TEE were assessed by 3 reviewers blinded to the clinical outcomes, following the echocardiographic protocol used in the RESPECT trial.^13^ The aortic distance was measured in the aortic valve short axis view (30°-60°), and the superior and inferior vena cava distances measured in the bicaval view (90°-120°) (Figure 1). A short aortic rim was defined as measuring <9 mm. The septum was described as mobile if total septal excursion was 10 to 15 mm; aneurysmal if total excursion was >15 mm or >10 mm in either the right or left atria; and with prominent aneurysm if the excursion was ≥20 mm. The tunnel height and length were measured in the aortic short axis and bicaval views. Bubble studies were considered positive if more than 1 bubble was seen in the left atrium within the first 3 cardiac cycles, at rest or with a Valsalva maneuver.

### Procedure

PFO closures were performed under conscious sedation and using fluoroscopic guidance alone with provisional use of intracardiac echo.^17^ In our practice, an Amplatzer device was chosen except for patients with nickel allergy. The device size (the vast majority 25 mm or 35 mm, based on the size of the right atrial disc) was chosen by the operator based on the preprocedural TEE images, focusing in particular to the mobility of the septum, with larger devices chosen for patients with a mobile interatrial septum, and height of the atrial septum to accommodate the disc size. Balloon sizing was not routinely performed. Patients were discharged on the day of the procedure if there were no complications. They had a follow-up visit with a transthoracic echocardiogram and a bubble study planned for 3 months after the procedure.

### Linkage to administrative databases for long-term outcomes

Long-term outcomes on the study population were obtained using linkage of the study clinical information to Ontario health administrative databases at ICES. The deterministic linkage was performed using Ontario health card numbers. Patients who were referred from other provinces and not followed in the province of Ontario and those with non-linkable records were excluded. ICES databases capture comprehensive information on emergency and inpatient admissions, outpatient physician visits, home and long-term care among others. Complete echocardiograms as part of long-term follow-up were not available in this registry, nor can individual complete medical records be accessed. Event review for etiology ascertainment and adjudication (presence of pericardial effusion at the time of atrial arrhythmia diagnosis, or device erosion that may not have been diagnosed) is therefore not available.

The follow-up period was from the day after hospital discharge until December 31, 2019. Following ICES policy to safeguard patient privacy, data fields with <6 patients were suppressed and reported as “1 to 5.”

### Statistical analysis

Baseline data are presented as counts and proportion or mean and standard deviation. Baseline characteristics and short-term outcomes were compared between groups using chi-square test for categorical variables and Student’s *t*-test for continuous variables. Long-term outcomes were reported using person-years and compared between groups using Poisson regression. Fine-Gray subdistribution hazards models were created for the outcomes of new-onset heart failure and new-onset atrial fibrillation, adjusting for age, sex, and baseline cardiovascular risk factors (diabetes and coronary disease for heart failure, and diabetes and heart failure for atrial fibrillation), and taking into account death as a competing event.^18^ Distribution of Schoenfeld residuals was used to evaluate the proportionality of subdistribution hazard assumption. The analyses were performed using SAS version 9.4; 2-sided *P* values of <0.05 were considered statistically significant.

The study was approved by the ethics review board of the University Health Network; a patient consent waiver was granted.

## Results

### Baseline demographics and echocardiographic parameters

Over the study period, there were 1,032 patients who underwent PFO closure and were included in the registry; of those, 625 had an Amplatzer PFO device implanted, and 324 (52%) had a preprocedural TEE images available for rim remeasurement (the majority of patients had preprocedural TEE but some were performed at outside institutions). The mean age was 49.8 years, and 56% were male. The most common indication for PFO closure was cryptogenic stroke (247, 72%) (Table 1). Patients with an aortic distance ≥9 mm were more likely to have a higher body mass index (28.1 vs 26.7 kg/m^2^, *P* < 0.02), diabetes (15% vs 7.6%, *P* = 0.035), heart failure (15.7% vs 4%, *P* < 0.001), and coronary disease (26% vs 16.8%, *P* = 0.044). They were more likely to have a non-stroke indication for PFO closure, such as platypnea-orthodeoxia or pacemaker/indwelling catheter.

**Table 1:** Baseline Characteristics

	Full Sample	Aortic Rim <9 mm	Aortic Rim ≥9 mm	*P* Value
	(N = 324)	(n = 197)	(n = 127)	
Year of procedure				0.50
2004-2008	50 (15.4)	27 (13.7)	23 (18.1)	
2009-2013	153 (47.2)	94 (47.7)	59 (46.5)	
2014-2017	121 (37.3)	76 (38.6)	45 (35.4)	
Age group in years				0.70
<40	74 (22.8)	46 (23.4)	28 (22.0)	
40-59	177 (54.6)	110 (55.8)	67 (52.8)	
60+	73 (22.5)	41 (20.8)	32 (25.2)	
Male	181 (55.9)	112 (56.9)	69 (54.3)	0.70
BMI (kg/m^2^)	27.3 ± 5.4	26.7 ± 4.8	28.1 ± 6.0	0.02
Comorbidities				
Prior/current smoker	92 (28.4)	50 (25.4)	42 (33.1)	0.10
Dyslipidemia	129 (39.8)	76 (38.6)	53 (41.7)	0.60
History of DVT/PE	38 (11.7)	22 (11.2)	16 (12.6)	0.70
Migraine	94 (29.0)	55 (27.9)	39 (30.7)	0.60
Atrial septal aneurysm^a^	99 (35.6)	67 (39.2)	32 (29.9)	0.10
Hypertension	126 (38.9)	73 (37.1)	53 (41.7)	0.40
Diabetes mellitus	34 (10.5)	15 (7.6)	19 (15.0)	0.035
Heart failure	28 (8.6)	8 (4.1)	20 (15.7)	<0.001
Chronic obstructive pulmonary disease	30 (9.3)	15 (7.6)	15 (11.8)	0.20
Coronary artery disease	66 (20.4)	33 (16.8)	33 (26.0)	0.04
Atrial fibrillation	23 (7.1)	11 (5.6)	12 (9.4)	0.20
Malignancy^b^	9-14	9 (4.6)	≤5	0.15
Renal failure^b^	≤5	≤5	≤5	0.97
RoPE score (modified^c^)	4.33 ± 1.79	4.40 ± 1.74	4.21 ± 1.85	0.40
Indication for closure				<0.001
Stroke	232 (71.6)	159 (80.7)	73 (57.5)	
Scuba diving^b^	11-16	≤5	11 (8.7)	
Platypnea-orthodeoxia syndrome/desaturation	15 (4.6)	6 (3.0)	9 (7.1)	
Pacemaker/indwelling catheter	22 (6.8)	8 (4.1)	14 (11.0)	
DVT/PE^b^	8 (2.5)	≤5	≤5	
Other	31 (9.6)	15 (7.6)	16 (12.6)	
Preprocedural medications				
Antiplatelets only	216 (66.7)	139 (70.6)	77 (60.6)	0.064
Anticoagulants only	20 (6.2)	6 (3.0)	14 (11.0)	0.004
Any antiplatelets or anticoagulants	288 (88.9)	182 (92.4)	106 (83.5)	0.013
Neither antiplatelets or anticoagulants	36 (11.1)	15 (7.6)	21 (16.5)	0.013
Transesophageal echocardiographic data				
PFO to aorta distance, mm	8.23 ± 3.81	5.84 ± 1.80	11.93 ± 3.07	<0.001
PFO to superior vena cava distance, mm	17.1 ± 6.5	17.0 ± 6.3	17.3 ± 6.7	0.70
PFO tunnel length, mm	10.2 ± 5.0	9.8 ± 4. 8	10.8 ± 5.3	0.07
PFO tunnel height, mm	4.4 ± 3.7	4.7 ± 3.7	3.8 ± 2.8	0.02
Interatrial septum thickness, mm	5.2 ± 2.5	5.3 ± 2.3	5.2 ± 2.5	0.70
Interatrial septum characteristics^d^				0.46
Non aneurysmal	260 (82.0)	153 (79.3)	107 (86.3)	
Aneurysmal	46 (14.5)	32 (16.6)	14 (11.3)	
Prominent atrial septal aneurysm^b^	8-13	8 (4.1%)	≤5	
Interatrial septal excursion, mm	8.6 ± 5.5	9.5 ± 5.6	7.2 ± 5.2	<0.001
Atrial septal defect in addition to PFO	15 (4.6)	9 (4.6)	6 (4.7)	0.99

Values are n (%) or mean ± SD.

BMI = body mass index; DVT = deep venous thrombosis; PE = pulmonary embolus; PFO = patent foramen ovale; RoPE = Risk of Paradoxical Embolism score.

a Information missing in 52 patients (16%) overall, with similar proportion in both aortic rim groups.

b Exact number cannot be reported due to small cells.

c RoPE score calculated without information on brain imaging as data were not available for all patients; the score presented therefore ranges between 0 and 9.

d Missing data in <10 patients.

The distribution of aortic distance is represented in Central Illustration. Most patients had a short aortic rim (<9 mm; 197/324, 60.8%), with a mean distance of 5.8 ± 1.8 mm in that group. The patients with a short aortic rim had a numerically higher incidence of atrial septal aneurysms (17% vs 11%) (Table 1), and a longer mean septal aneurysmal excursion (9.5 vs 7.2 mm, *P* < 0.001). The superior vena cava rim length was similar in both groups, measuring a mean of 17 mm. Five percent of patients in both aortic distance groups had an associated atrial septal defect.

**Central Illustration:**
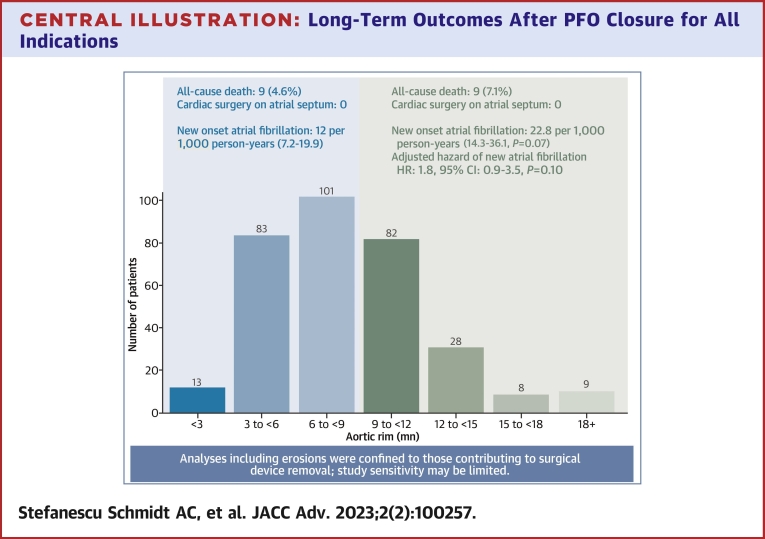
**Long-Term Outcomes After PFO Closure for All Indications** Long-term outcomes in the short (<9 mm) and longer (≥9 mm) aortic rim groups. Hazard of new atrial fibrillation diagnosis in long-term follow-up is adjusted for baseline age, sex, pre-existing heart failure and diabetes. Indications for PFO closure in this cohort include cryptogenic stroke, treatment of platypnea orthodeoxia syndrome, prophylaxis of decompression illness for scuba diving, and prevention of paradoxical embolism in selected patients with permanent indwelling lines or pacemaker wires.

### Procedural characteristics and in-hospital outcomes

A 35 mm device was implanted in most patients in both groups (64% in the short vs 59% in the aortic rim ≥9 mm group) (Table 2). There were no cases of atrial arrhythmias, major vascular complications, or bleeding events after the procedure. Fewer than 6 patients in both groups were treated intraprocedurally for paroxysmal atrial tachyarrhythmias, with antiarrhythmic drugs or cardioversion.

**Table 2:** Procedural Outcomes

	Full Sample (N = 324)	Aortic Rim <9 mm (n = 197)	Aortic Rim ≥9 mm (n = 127)	*P* Value
Device size (mm)				0.43
35	200 (61.7)	125 (63.5)	75 (59.1)	
25 or 30^a^	124 (38.3)	72 (36.5)	52 (40.9)	
Procedural success	324 (100)	197 (100)	127 (100)	
In-hospital outcomes				
In-hospital death	0	0	0	
Myocardial infarction	0	0	0	
Stroke	0	0	0	
Dialysis	0	0	0	
Pericardial effusion	≤5^b^	0	≤5^b^	0.40
Major vascular complications	0	0	0	
Intra-procedural arrhythmia requiring treatment	≤5^b^	≤5^b^	0	0.20
Postprocedural new-atrial fibrillation or flutter	0	0	0	
Need for pacemaker	0	0	0	
Major access bleeding	0	0	0	

Values are n (%) or n.

a 30-mm devices used in 2.2% of all patients.

b Exact number cannot be reported due to small cells.

### Long-term outcomes

Over a median of 7 years of follow-up (IQR: 4-10 years), there were no cases of device embolization or erosion requiring cardiac surgery in either group (Table 3). There were 9 all-cause deaths in each group (4.6% in the short rim group vs 7.1% in the rim ≥9 mm, *P* = 0.30). The incidence of stroke overall was 2.2%, and that of stroke or possible transient ischemic attack was similar in both groups (6.3% in those with aortic rim ≥9 mm vs 5.1% in those with rim <9 mm, *P* = 0.60; incidence rate ratio of 1.1 (0.3-5), *P* = 0.90). The incidence of new-onset atrial fibrillation was numerically higher in the group with aortic rim ≥9 mm (12.0 vs 22.8/1,000 person-years, with incidence rate ratio of 1.9 (1-3.8), *P* = 0.07).

**Table 3:** Long-Term Outcomes

	Full Sample	Aortic Rim <9 mm	Aortic Rim ≥9 mm	*P* Value
	(N = 324)	(n = 197)	(n = 127)	
Follow-up time	7 (4-10)	7 (4-9)	7 (4-10)	0.60

	Event Rates Per 1,000 PY (95% CI)			IRR (95% CI)	
All-cause mortality	7.8 (4.9-12.3)	6.5 (3.4-12.4)	9.7 (5.1-18.7)	1.5 (0.6-3.8)	0.40
Surgery on atrial septum (PFO/ASD)	0	0	0	-	-
Cardiac surgery for foreign body removal	0	0	0	-	-
New-onset atrial fibrillation	16.2 (11.5-22.7)	12.0 (7.2-19.9)	22.8 (14.3-36.1)	1.9 (1-3.8)	0.067
New-onset heart failure	10.2 (6.6-15.6)	8.4 (4.7-15.2)	13.2 (7.1-24.5)	1.6 (0.7-3.7)	0.30
Stroke	3.0 (1.5-6.4)	2.9 (1.1-7.7)	3.3 (1.1-10.1)	1.1 (0.3-5)	0.90
Stroke/TIA	8.0 (5.1-12.8)	7.3 (2.4-10.7)	9.1 (4.6-18.2)	1.2 (0.5-3.1)	0.70
Major bleeding	0.9 (0.2-3.5)	1.4 (0.4-5.8)	0	-	-

Values are median (IQR) unless otherwise indicated.

ASD = atrial septal defect; CI = confidence interval; IRR = incidence rate ratio; IQR = interquartile range; PFO = patent foramen ovale; PY = person-years; TIA = transient ischemic attack.

After adjusting for age, sex, pre-existing heart failure and diabetes, the risk of new-onset atrial fibrillation in the group with an aortic rim ≥9 mm was not different between the groups (HR: 1.76, 95% CI: 0.89, 3.5, *P* = 0.10). Similarly, the incidence of new heart failure was not significantly different between aortic rim groups (incidence rate ratio: 1.6, 95% CI: 0.7, 3.7, *P* = 0.30), including after adjusting for age, sex, baseline coronary disease, and diabetes (HR: 1.63, 95% CI: 0.69, 3.9, *P* = 0.30).

## Discussion

In this large single-center series of patients undergoing percutaneous PFO closure with the Amplatzer PFO Occluder, the procedure was successful as defined in our study and safe over a median of 7 years of follow-up, regardless of aortic distance on baseline TEE. More than half of the cohort of patients presenting for PFO closure had an aortic rim <9 mm, and in that group, there were no device erosions requiring cardiac surgery over 1,394 patient-years of follow-up.

Patients with a longer aortic rim were more likely to have a higher body mass index, and more likely to have a history of congestive heart failure; previous autopsy studies have similarly reported an association between longer aortic rim and higher body surface area.^9^ Conversely, patients with a shorter aortic rim had a trend toward more frequent atrial septal aneurysms. There were no significant differences in all-cause mortality, stroke or transient ischemic attack in long-term follow-up between groups. After adjusting for baseline risk factors (such as diabetes, coronary disease, and pre-existing heart failure), there were no significant differences in risk of new-onset atrial fibrillation or heart failure. The incidence of postprocedural atrial fibrillation in long-term follow-up (16.2/1,000 person-years, 95% CI 11.5, 22.7) is similar to recently published administrative data^19^ and meta-analyses.^7^^,^^20^ Previous analyses have suggested that larger devices are associated with an increased risk of postprocedural atrial fibrillation.^21^ The incidence of all-cause death in this cohort may be related to the older population in this study and more frequent comorbidities (22% of patients over the age of 60 overall, with 16% with heart failure, 26% with coronary artery disease, and 15% with diabetes in the longer aortic rim group); it is similar to what has been reported in similar age groups.^22^^,^^23^

Our study has detailed preprocedural echocardiographic analysis, adding to the previous case reports and surveys that report a similarly low risk of erosion after PFO closure. In a retrospective survey of 18 centers in Europe and the U.S. published in 2011,^24^ 38 (0.3%) device explantations were reported among 13,736 PFO closures (0.21% of the implanted Amplatzer devices). Of note, many of those centers reported using the Amplatzer atrial septal occluder (ASO) or Cribriform atrial septal defect (ASD) device, as the PFO device was only approved by the Food and Drug Administration in 2016; the ASO device is stiffer and has a larger self-centering waist, as opposed to the PFO device which has a thin, short, flexible waist that allows the discs to move independently. Only 2 devices were explanted for cardiac perforation, of which one was an Amplatzer PFO 25 mm device (0.01%) late after the procedure, and one a CardioSEAL device (NMT Medical) (0.05%) that was explanted just hours after the procedure. Two additional Amplatzer devices (type not specified) were removed due to pericardial effusion (which may have represented an erosion, procedural complication, or inflammatory reaction). Explantations due to residual shunt were more common, seen in 12 patients (0.09%; 4 with Amplatzer, 0.04%), in which device malposition or an atrial septal injury was noted and required surgical removal rather than another percutaneous closure.

As of 2019, there were over 100,000 implantations of the Amplatzer PFO device.^25^ In the 3 cases reported in the literature of erosions linked to the Amplatzer PFO device, the aortic rims were normal in 1,^16^ and unknown in the other 2,^14^^,^^15^ with all 3 cases demonstrating erosions through the atrial wall. The size of the atria and the distance from the PFO to the atrial wall (and subsequent choice of device size), as well as the final position of the device, appear to be more relevant to the risk of erosion than the aortic rim. Reviewing the safety events reports to the Food and Drug Administration Manufacturer and User facility Device Experience (MAUDE) database for Amplatzer PFO devices through March 31, 2021, there were 3 reports of device erosion requiring surgical removal for the 35 mm device. One patient had injury to the aorta, and the second was reported to have atrial and aortic injury repaired with pledgeted sutures but the device was left in place; there are no details for the third. There were no reported device erosions for the 30 mm, and 3 reports of erosion for the 25 mm device (all 3 injuries to the right atrial wall). The 6 patients who had reported erosions presented with dyspnea or chest pain and were found to have pericardial effusions on echocardiogram; atrial or aortic injury was confirmed at the time of operation for device removal. Three patients presented within 5 days from the procedure, and another 2 within a year. In our series with longer follow-up, there were no patients who required cardiac surgery for an atrial septal repair. We may not, however, have captured patients who suffered sudden cardiac death events or expired before surgery could be performed.

The risk of erosion from septal occluder devices was raised in a review of adverse events after implantation of the Amplatzer ASD Septal Occluder device; in the initial series of 28 patients, all erosions were noted in the dome of the atrium, near the aorta, and 89% had a deficient aortic root on review.^26^ Practice has moved away from oversizing devices more than 2 mm from the balloon-stretched size of the ASD (which was more commonly done at the time of that study), and focused on an adequate aortic rim size. The ASO device in contemporary series has been shown to be implanted safely in patients with deficient aortic rims (<5 mm) acutely (from the American College of Cardiology National Cardiovascular Data Registry IMPACT multicenter registry^27^) and with a high success rate (99%) and no adverse events over 24 months of follow-up in a recent single-center study of 400 patients.^28^

The original Instructions for Use of the PFO device were based on the initial experience with the Amplatzer Septal Occluder, though the characteristics of the PFO device make erosions significantly less likely. In particular with PFO devices, a higher incidence of erosion has not been demonstrated with larger devices (35 mm), while a higher incidence of embolization (either during or after the procedure) is known, in particular in patients with an aneurysmal atrial septum. In our experience, as previously reported,^17^ there was one device embolization during the procedure in the setting of a hypermobile atrial septum, treated with surgical device removal and PFO closure. There were no other cases of device embolization or erosion.

### Study Limitations

We present a single-center analysis, with long-term follow-up from an administrative database, introducing the potential for misclassification bias; the robust patient linkage and completeness of data in follow-up have been previously described. Chart abstraction was done retrospectively, and some data is missing; full echocardiographic data were not available for all patients. Echocardiograms in long-term follow-up were not available, nor was it possible to review charts at the time of death to determine if cause of death, or if there was a possible pericardial effusion or device erosion that may not have been detected. Pericardial effusions that may be associated with device erosion (or a non-device related cause, or inflammatory or allergic reaction) were also not captured, and may limit the sensitivity of this study. We choose to focus on severe erosions requiring device surgical removal; device erosions associated with sudden death would not be captured in this registry. As the incidence of erosion is low in the population overall (previously reported at 0.2%,^5^ though as low as 0.02% in other series^24^), we would expect to see only 1 event, or perhaps less, in a cohort of our size. If a short aortic rim carried a clinically relevant higher risk of erosion, we would anticipate a noticeable number of events as more than half of the implanted patients in our cohort had a short aortic rim. Our long duration of follow-up was designed to capture both acute and late events, as has been previously described.

## Conclusions

Percutaneous closure of a PFO using the Amplatzer PFO Occluder was performed safely in a large single-center series, including in a majority of patients with a PFO to aortic distance measuring <9 mm, without surgical device removal or repair for erosions in long-term follow-up. Our experience is consistent with the very rare reports of erosion requiring explantation of <1:1,000.

## Funding support and author disclosures

Dr Horlick is a consultant for Edwards, Medtronic and Abbott; and he has received research grants from Abbott. The Structural Heart Disease program at University Health Network receives educational support and fellowship funding from Abbott, Edwards and Medtronic, and is a participant is clinical trials for devices from Abbott, Edwards and Medtronic. Drs Osten and Benson are consultants for Abbott, Edwards, and Medtronic. Edwards, Medtronic, and Abbott were not involved in planning or execution of this analysis. Dr Ephrem received speaking honorarium from Zoll. Zoll was not involved in planning or execution of this analysis. This work is supported by the Peter Munk chair in Structural Heart Disease Intervention, and in part by an investigator-initiated research grant from 10.13039/100001316Abbott on long-term outcomes of PFO transcatheter closure in adults. ICES is supported in part by a grant from the 10.13039/501100000226Ontario Ministry of Health and Long-Term Care. The opinions, results, and conclusions are those of the authors and no endorsement by the Ministry of Health and Long-Term Care or by ICES is intended or should be inferred. Parts of this material are based on data and information compiled and provided by Canadian Institute for Health Information (CIHI). However, the analyses, conclusions, opinions, and statements expressed herein are those of the author, and not necessarily those of CIHI. Dr Lee is the Ted Rogers Chair in Heart Function Outcomes, a joint Hospital-University Chair of the University Health Network and the University of Toronto. Dr Horlick is supported by the Peter Munk Chair in Structural Heart Disease Intervention. All other authors have reported that they have no relationships relevant to the contents of this paper to disclose.

PERSPECTIVES **COMPETENCY IN PRACTICE-BASED LEARNING:** Use of a large administrative database with long-term follow-up, combined with a detailed institutional database, allowed study of long-term outcomes to capture rare events (device erosion after transcatheter PFO closure).

**TRANSLATIONAL OUTLOOK:** Continued analysis of long-term outcomes after transcatheter PFO closure, in particular in young patients with a normal life expectancy, is needed, in particular if the indications for PFO closure are expanded.
